# Updating Clear Cell Renal Cell Carcinoma (a Tribute to Prof. Ondrej Hes)

**DOI:** 10.3390/cancers14163990

**Published:** 2022-08-18

**Authors:** Claudia Manini, José I. López

**Affiliations:** 1 Department of Pathology, San Giovanni Bosco Hospital, 10154 Turin, Italy; 2 Department of Sciences of Public Health and Pediatrics, University of Turin, 10124 Turin, Italy; 3 Unit of Biomarkers, Biocruces-Bizkaia Health Research Institute, 48903 Barakaldo, Spain

This Special Issue provides an insight into critical issues concerning clear cell renal cell carcinomas (CCRCCs), reflecting the recent level of intricacy reached by renal oncology. The collection includes nineteen papers (nine articles, eight reviews, one perspective, and one commentary) which deal with contemporary diagnostic, prognostic, and therapeutic aspects of this tumor. Moreover, this Special Issue aims to provide a humble and sincere homage to the memory of Prof. Ondrej Hes, a worldwide referential Czech pathologist in renal cancer, who passed away unexpectedly on 2 July 2022 at the age of 54. We are honored to have two contributions co-authored by him (refs. [1,2]) in this collection.

Manini et al. [3] focus on tumor sampling as a cornerstone to scrutinize the complexity of intratumor heterogeneity (ITH) in CCRCC. Based on the recent molecular findings of tumor regionalization [4], the authors propose focalizing tumor sampling on peripheral zones, where ITH is expected to be the highest. Conversely, the tumor interior, where metastasizing subclones develop, is more homogeneous.

Sequeira et al. [5] show that a specific pattern of miRNA expression characterizes CCRCC with a sensitivity of 74.78%. This pattern includes hsa-miR-126-3p and hsa-miR-200b-3p levels. The authors conclude that this minimally invasive test may be useful to detect CCRCC in the early stages of tumor development.

Gopal et al. [6] review the current advances and future directions of the use of radiogenomics in the management of CCRCC. The authors update the issue and stress the promising correlation found between imaging features and gene expression patterns in several neoplasms, particularly in CCRCC.

Vano et al. [7] review the first-line treatment options in metastatic CCRCC. They state that a strategy based on the International Metastatic Database Consortium is currently recommended with either pembrolizumab and axitinib, cabozantinib and nivolumab, or levantinib and pembrolizumab given as the first-line treatment for all patients. Additionally, patients with an intermediate or poor risk should be treated with nivolumab and ipilimumab. They indicate that several issues, such as PD-L1 status, are unresolved and deserve further analyses. Thus, making therapeutic decisions based on a reliable immunohistochemical detection of the PD-L1 is still a matter of controversy [8].

Larrinaga et al. [9] compare the plasma and tissue expression of PD-1 and PD-L1 in a series of 89 CCRCCs. This unprecedented analysis yielded some significant results, for example, the plasmatic levels of both proteins were lower in CCRCC patients than in the controls. The study also confirms that the high expression of PD-1 and PD-L1 in tumor tissue was associated with tumor grade, size, and tumor necrosis. While PD-1 was associated with tumor stage (pT), PD-L1 was associated with metastases. The combination of plasmatic and tissue positivity increased the level of significance to predict the prognosis of these patients.

Several contributions deal with the ever-changing landscape of therapies and resistances to therapies occurring in these tumors. Three clinical reviews [10,11,12] and one article analyzing sunitinib resistance in CCRCC cell lines [13] revisit this particularly important issue.

Ballesteros et al. [10] focus on the molecular mechanisms of resistance to immunotherapy and antiangiogenic drugs. Resistance associated with tyrosine-kinase inhibitors include molecular mechanisms related to hypoxia, the angiogenic switch, epithelial-to-mesenchymal transition, the activation of bypass pathways, the lysosomal sequestration of tyrosine kinase inhibitors, non-coding RNAs and single-nucleotide polymorphism, and the tumor microenvironment. Among the pathways associated with resistance to immune checkpoint inhibitors, the authors analyze interferon gamma signaling, Wnt/β-catenin, MAPK, PI3K/AKT/mTOR, cell cycle checkpoint, the loss of major histocompatibility complexes I and II, and the tumor microenvironment.

Angulo et al. [11] analyze the epigenetic landscape of CCRCCs. Thus, abnormal DNA methylation, methyl-binding proteins, post-translational histone modifications, miRNAs, long non-coding RNAs, and RNA methylation are thoroughly reviewed. Furthermore, the authors revise the epigenetic-based therapeutic opportunities for CCRCCs and the caveats and limitations of these treatments.

Kim et al. [12] update the immune landscape and the immunotherapy opportunities of CCRCCs, i.e., cytokine-based immunotherapy, tyrosine kinase and mTOR inhibitors, and immune checkpoint inhibitors. The authors also focus on single-cell genomics to analyze the tumor microenvironment.

Sunitinib is a standard first-line treatment for metastatic CCRCCs [14]. Armesto et al. [13] have identified miRNA:target interactions involved in sunitinib resistance using three CCRCC cell lines (786-O, A498, and Caki-1). They have demonstrated that the use of in vitro models of sunitinib resistance, combined with an integrated approach of miRNA and gene expression, can identify divergent mechanisms of resistance with potential benefit for patients.

Paderi et al. [15] retrospectively evaluate the immune-related adverse effect of nivolumab and ipilimumab in 43 patients with metastatic renal cell carcinomas, 36 of them being CCRCCs. They conclude that adverse effects, such as thyroid dysfunction and cutaneous reactions, were associated with longer progression-free survivals and that patients that experienced more than one adverse effect presented a better response to treatment. Endocrine disorders, notably thyroid toxicities, must be taken into account since they present clinically with vague symptoms and unclear clinical pictures. The effect of nivolumab on the PD-1 expression in a culture model of CCRCC has been analyzed by Stenzel et al. [16]. They conclude that data obtained from ex vivo tissue slice culture may predict patient response to nivolumab. The influence of molecular subtypes based on genomic and transcriptomic features in the responsiveness of metastatic CCRCCs to immune checkpoint inhibitors has been reviewed by Jee et al. [17].

Mattila et al. [18] analyze the existing prognostic features and prediction models for localized CCRCCs, a growing group of tumors with an unpredictable clinical course. They conclude that prognostic factors and prediction models may help evaluate the risk of recurrence after surgical resection in localized CCRCCs, which would reduce follow-up imaging in low-risk cases. Additionally, better prediction models would help select patients for adjuvant trial therapies.

Lipidomic analysis adds interesting information in normal and neoplastic kidneys. Molecular histology has recently been profiled in non-tumor kidney tissue using the mass spectrometry of lipids [19]. Data obtained in this study demonstrate that up to seven lipidic patterns correlate with different parts of the nephron, allowing one to distinguish characteristic lipidic fingerprints in different individuals. The lipidomic analysis performed in samples from 12 CCRCCs has demonstrated the overexpression of stearoyl-CoA desaturase-1 (SCD-1) induced by the hypoxic microenvironment [20] which is characteristic of this neoplasm. The authors have detected a particular lipidomic composition involving SCD-1 in the center of CCRCC which in turns depends on the high hypoxic status found at this level. They conclude that SCD-1 may be a potential target in future treatments of these tumors. Other authors have detected that metastasizing clones of CCRCCs are located in the tumor’s center [4], where hypoxia is high and the struggle for survival is fierce. 

The molecular heterogeneity in paired primary and metastatic samples of CCRCCs has previously been analyzed [21,22]. Prochazkova et al. [1] have studied the mutational variability between primary CCRCCs (four cases) and their multiple pulmonary metastases (nine metastases in total). The authors conclude that all the cases studied displayed high mutational variability not only when comparing the primary tumors, but also among the metastases themselves. These findings confirm the previous analyses which stress the high inter- and intratumor variability in most CCRCCs, a feature of critical importance when making therapeutic decisions for patients.

Recent studies have shown that the angiogenic type of CCRCC is linked to *PBRM1* gene loss [23]. In this Special Issue, Saiga et al. [24] correlate the immunohistochemical expression of PBRM1 with specific architectural and vascular patterns in CCRCCs. The authors found that endothelial expression tends to be lost in cases with low PBRM1 expression. Previous studies of the same research group have demonstrated that a vascularity-based architectural classification of CCRCC has prognostic implications [25].

Khaleel et al. [26] analyze the translation between the radiologic phenotype and the underlying genotype available in the current radiogenomics literature of CCRCCs, reviewing PubMed, Medline, Cochrane Library, Google Scholar, and the Web of Science databases. Most studies use computed tomography images and the most common genomic mutations of CCRCC (VHL, PBRM1, BAP1, SETD2, and KDM5C) for such translation. They conclude that the field is promising but further studies are needed to implement this approach in clinical practice.

Roldán et al. [27] define a gene-expression-based signature in CCRCCs with prognostic implications based on a whole-transcriptome profiling of 26 cases. They found a total of 132 genes related to prognosis; however, following a Cox analysis, a nomogram including *CERCAM*, *MIA2*, *HS6ST2*, *ONE-CUT2*, *SOX12*, and *TMEM132A* genes, together with pT stage, tumor size, and ISUP grade, has been generated. The authors conclude that this nomogram discriminates between two different groups of CCRCCs with different probabilities of recurrence and predicts cancer-specific survival.

A commentary in this CCRCC Special Issue refers to the urologist’s perspective of the so-called multilocular cystic renal neoplasm of low malignant potential [2]. The clinical, radiological, and pathological findings as well as the therapeutic management are reviewed. They conclude that this entity is a lesion with excellent prognosis in which a conservative nephron-sparing treatment, if technically possible from the surgeon’s perspective, should be performed.
